# A case study of using community-based consensus methods to facilitate shared decision-making among a spinal cord injury network

**DOI:** 10.3389/fresc.2024.1335467

**Published:** 2024-02-16

**Authors:** Emily E. Giroux, Peter Athanasopoulos, Shane N. Sweet, Heather L. Gainforth

**Affiliations:** ^1^ Centre for Health Behaviour Change, School of Health and Exercise Sciences, University of British Columbia Okanagan, Kelowna, BC, Canada; ^2^ International Collaboration on Repair Discoveries (ICORD), University of British Columbia, Vancouver, BC, Canada; ^3^ Department of Public Policy and Government Relations, Spinal Cord Injury Ontario, Toronto, ON, Canada; ^4^ Department of Kinesiology & Physical Education, McGill University, Montreal, QC, Canada

**Keywords:** spinal cord injury (SCI), consensus methods, shared decision-making, research partnership, Delphi methodology, inclusive research, integrated knowledge translation (IKT), policy-making

## Abstract

Spinal cord injury (SCI) research and policy decisions are rarely made in partnership with people with SCI, making them less relevant, applicable, and used by those whom the decisions are intended to support. Across disciplines, consensus methods have been promoted as a viable solution for supporting shared research and policy-based decision-making. In this paper, we describe a partnered approach between academic researchers and the Ontario SCI Alliance, a non-profit, SCI community mobilization network to co-develop and co-disseminate a community-based consensus exercise. The community-based consensus exercise included two modified Delphi surveys and one in-person retreat. The partnership's goal with this exercise was to facilitate shared decision-making for the development of their upcoming strategic plan. We then interviewed partners and participants from the Delphi and in-person retreat to discuss successes, challenges, and lessons learned from the exercise. Survey 1 was disseminated to over 2,500 members of the Ontario SCI community and received 374 responses (276 coming from people with SCI). Survey 2 had 118 responses, with 87 coming from people with SCI. The retreat had 73 attendees, including people with SCI, family/friends of people with SCI, clinicians, researchers, and SCI community and research organization staff/volunteers. The retreat included a presentation of the survey results, a clinician/researcher panel, and externally-facilitated working groups. All survey responses and retreat materials were synthesized. Using the synthesized feedback, the Ontario SCI Alliance was able to implement several changes for the Ontario SCI community, including higher-quality primary care experiences (reduced wait times, more accessible examining rooms), the development of a wound care strategy with the Ontario government, and an advocacy campaign for public coverage for catheters and urinary care supplies. From the five interviews conducted, five themes were co-constructed regarding the successes, challenges, and lessons learned from the exercise: (1) Inclusion, Diversity, Equity, and Accessibility; (2) Partnership; (3) Design Considerations; (4) Transparency and Clarity in Communication; and (5) Sustainability. Findings from this community case study demonstrate the feasibility of conducting a community-level consensus exercise among an equity-deserving group while providing detailed guidance for how to ensure future research and policy-based decision-making is shared across diverse knowledge users.

## Introduction

Paralysis is often viewed as the primary damaging outcome of a spinal cord injury (SCI). However, people with SCI experience complex physical and psychological complications (e.g., loss of bowel and bladder function, decreased skin integrity, and reduced feelings of independence) that someone without a SCI cannot understand (1). Beyond these complications, people with SCI have been marginalized and experience inequities (2, 3). People with SCI are seldom in positions to influence research, practice, and policy decisions, even when the decisions directly impact them. Indeed, researchers and policymakers have begun approaching people with SCI to be involved in research and policy initiatives. However, while engaging, some researchers and policymakers have been faulted of tokenism, which occurs when equity-deserving groups have limited decision-making power, promoting a false sense of representation and endorsement (4, 5).

Strategies for meaningfully incorporating multiple and diverse perspectives of SCI lived experience must be prioritized to combat tokenism and promote equitable decision-making. Across disciplines, consensus methods including the Delphi method (6), Nominal Group Technique (7), and Deliberative Dialogue (8) have been used to consider multiple perspectives in decision-making. As such, consensus methods may be valuable in improving decision-making with SCI communities.

Researchers have promoted consensus methods as promising for developing relevant and impactful research agendas (9, 10), while also advocating for equity-deserving groups to be involved in determining policies. Using consensus methods in a policy context can promote inclusion by ensuring decisions are informed by individuals directly impacted by the decisions (11, 12). When policy decisions are made *with*, and not *for* equity-deserving groups, there can be more confidence in the effectiveness and potential impacts of the policy. A critical step in advocating for more equitable policy-based decision-making is demonstrating the feasibility and impact of using consensus methods to address this issue. For SCI communities particularly, the Delphi method may be valuable given its previous use in policy contexts (13, 14) and unique features that promote inclusion.

Traditionally, Delphi methodology has been understood as a formal and systematic way for “experts” in a topic to arrive at consensus that involves the iteration and distribution of surveys in rounds until consensus is reached (6, 15). Delphi methodology has distinct features that can help address issues that SCI communities may face when convening to make decisions. Being able to complete surveys on your own time and privately, can help people who may lack time due to self-care or unforeseen health issues, face geographical or accessibility barriers, or feel intimidated by contradictory opinions and power dynamics (6, 15). Its use has extended to SCI peer mentorship research (ranging from 45 to 84 participants with SCI-lived experience) and in-patient rehabilitation best practices (one participant with SCI-lived experience) (16–18). While these Delphi studies have expanded the meaning of “expert” to extend beyond academic and clinical experts, to our knowledge, the use of a Delphi to facilitate community member engagement in SCI policy-making at the provincial level has yet to be explored.

Given its quantitative nature, the Delphi is one of the most commonly used consensus methods across disciplines (10). Yet, many published Delphi methods include limited reporting of the informal and internal processes to develop and carry out a Delphi, making it challenging for researchers and communities alike to learn about and subsequently use consensus methods in their work (10). To promote reporting transparency and explore the application of the Delphi method to communities, this paper presents a case study of using a community-based Delphi consensus method to support the Ontario SCI Alliance, a SCI mobilization network, in determining research and policy initiatives to fund in their organizational strategic plan. This paper aims to demonstrate the feasibility and impact of a community-based consensus method by describing:

1. The development and dissemination of the method.
2. The successes, challenges, and lessons learned from the perspectives of individuals involved in developing, disseminating, and/or participating in the method.

## Context

The Ontario SCI Alliance (Alliance) was developed under the leadership of Spinal Cord Injury Ontario (SCIO) and the Ontario Neurotrauma Foundation. The Alliance has over 250 members, including over 70 organizations, and a readership of over 10,000 Ontario SCI community members. Throughout 2017, the Alliance hosted Summit meetings to bring together researchers, clinicians, policymakers, and people with SCI to address SCI clinical care, research, and policy issues. Twelve meetings took place, each focused on one of the following domains: bladder management, neuropathic pain, pressure injuries, primary care/community supports, acute interventions, wheeled mobility, self-management, cardiovascular integrity, emotional well-being, walking, upper limb integrity, and sexual health. For each domain, the Alliance worked with expert researcher clinicians to synthesize meeting proceedings with pre-existing evidence. After reviewing the syntheses, the Alliance deemed four key topics urgent to address: primary care/community supports, neuropathic pain, bladder management, and pressure injuries. The Alliance then revisited proceeding syntheses for the four selected domains (19–22) and determined 34 strategies for consideration in their upcoming 3-year strategic plan. If included, strategies would have effort, time and resources dedicated to their implementation.

The Alliance expressed the need to meaningfully include their membership when deciding on strategies to implement. The Alliance's Executive Director (PA) contacted a previous academic research partner (HG) to help achieve this goal. PA and HG had previously partnered on a series of research projects on disseminating SCI Physical Activity Guidelines across Ontario (23–27). HG applied for funding to support a trainee (EG) to co-lead the new partnership's activities, and invited SS to build and think through the study's methodological components. Through discussions within the partnership, it was determined that co-developing a large-scale, community-based consensus method informed by Delphi methodology could help the Alliance meet its goal.

### Development and dissemination of the community-based consensus method

**Figure 1:**
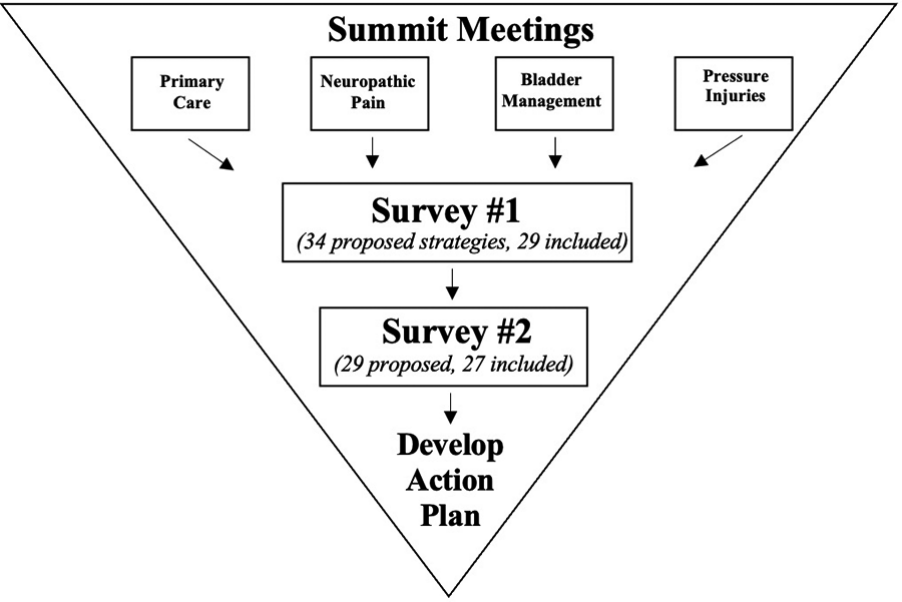
Visual representation of community-based consensus exercise.

The community-based consensus method included one initial survey (Survey 1), one subsequent survey (Survey 2) and a one-day in-person retreat. Both surveys were hosted on SimpleSurvey™ Software, and the retreat occurred at the Hart House in Toronto, Canada. Figure 1 outlines each stage and associated timelines.

The partnership adopted an integrated knowledge translation approach, meaning that all partners (three researchers, one community partner with SCI lived experience and decision-making power with the Ontario SCI Alliance) were meaningfully engaged throughout the research process. Supplementary File S1 includes a detailed account of the partnership's development and activities.

### Survey 1 development

To create survey content, the 34 pre-determined strategies were organized by domain: primary care and community supports *(n = 10 items)*; neuropathic pain *(n = 8)*; bladder management *(n = 8);* and pressure injuries *(n = 8)*. Each domain was given a page in the survey. Each page began with a brief definition and description of the domain, followed by the strategies for that domain. While traditional Delphi methods are designed for “experts” in a topic, we included definitions and descriptions to ensure *all* respondents could understand the items presented. Descriptions for each strategy were written at a Canadian grade 8 reading level to enhance comprehension of survey content and extend the idea of “expertise” beyond education level. Each strategy was paired with an 11-point Likert scale, where participants could indicate their level of agreement with implementing each strategy (0 = strongly disagree with implementing the strategy; 10 = strongly agree with implementing the strategy). Strategies were randomized within each domain. After presenting all strategies for a domain, participants were given an open textbox to share insights about anything that may not have been included in the strategy list. Open-ended questions are uncommon for Delphis but allowed individuals who could not attend the Summit meetings to share their unique and important perspectives. After survey completion, a separate online link was sent to participants, allowing them to provide consent and contact information for future survey rounds.

Upon completing the initial draft of Survey 1, PA shared the survey with 14 Alliance members with SCI lived experience, and/or expertise in research and/or policy. Sharing the survey with members outside of the immediate partnership allowed the survey to be further refined for accuracy, clarity, and acceptability. Once proposed changes were implemented, Survey 1 was piloted with four members of the Ontario SCI community, and minor refinements were made to create the final version.

### Survey 1 dissemination

SCIO staff were responsible for survey dissemination, including any communications associated with the survey (e.g., reminders to complete the survey, social media advertisements). Surveys were disseminated through e-mailing SCIO and Alliance membership databases, website advertisements, and Twitter/Facebook postings. Participants were given two months to complete the survey and received three reminders to complete the survey two weeks, one week, and one day before the survey closed.

### Survey 1 analysis

Aligning with traditional Delphi methods, Survey 1 results informed the development of Survey 2. e.g., analyzed the initial survey responses within one week of closing the survey. The mean score, highest score, and lowest score were calculated for each strategy. Strategies were only included in Survey 2 if they met one of two *a priori* consensus criteria: (a) had a mean score greater than or equal to 8.0 *or* (b) had two-thirds of participants rate the strategy as an 8.0 or above (16).

### Survey 2 development, dissemination, and analysis

Survey 2 was formatted identically to Survey 1, with the primary difference being that the descriptive statistics (i.e., mean score, highest score, lowest score) and consensus values for each strategy were presented beside each item. With this information, participants are again asked to indicate their level of agreement with each strategy against the same 11-point Likert scale. The link to complete Survey 2 was only e-mailed to participants who completed Survey 1 *and* indicated interest in participating in future consensus surveys. Respondents were given 2 months to complete Survey 2 and were provided with the same three completion reminders prior to the survey closing. At the end of the survey, participants were asked to indicate if they were interested in participating in the in-person retreat, where survey results would be discussed and incorporated into working-group activities. This iterative process of development and analysis was repeated until participants reached consensus on all strategies.

### Retreat

To promote meaningful engagement beyond survey completion, PA suggested hosting a one-day in-person retreat within the Alliance's previously scheduled annual meeting. Strategically combining the events ensured that the Alliance was being considerate of their memberships’ other commitments and priorities. The retreat consisted of three key events: (1) presentation of survey results, (2) expert panel discussion, and (3) facilitator-led working groups. Seventy-three people, including people with SCI lived experience, researchers, policymakers, and clinicians attended the retreat. Binders with summarized information from the presentation and panel discussion were provided to attendees to be used throughout the day.

#### Presentation and panel discussion

EG created and delivered a presentation to summarize survey findings: respondent demographics, strategies that did/did not meet consensus, and responses from open-ended questions. Attendees were then able to ask EG questions about the surveys. Following the presentation, PA moderated a panel with the five researcher clinicians who synthesized the evidence used to determine strategies for the survey. Attendees could also ask panel members questions after the discussion, which provided a more comfortable space for people with SCI lived experience to ask questions to individuals they would not normally have the opportunity to ask. Lunch took place after the panel discussion, giving attendees time to digest the information from the morning, and informally network with other attendees.

#### Facilitated working groups

After lunch, an externally-hired facilitator led all attendees in group-based brainstorming activities that incorporated the survey results. There were eight working groups, with two tables dedicated to each domain. Seating arrangements were determined *a priori*, to ensure tables were a heterogeneous mix of clinicians, researchers, people with SCI, community organization staff, and policymakers. Using pre-determined questions co-developed by PA and the external facilitator, the external facilitator encouraged each working group to collectively engage in critical thinking and discussion. The questions asked included:

• What are the proposals that should be the focus of engagement work by the community?
• What are the things to remember to engage our community over the next 3 years in this work?
• What are the things we should avoid when engaging the community in this work?
• What will the impact of our collective work be at the end of the 3 years?

Each working table was assigned a note-taker and a sub-facilitator to keep the table on-topic and ensure equitable sharing. Facilitators and note-takers were provided with two worksheets (one page with instructions, one page with each question and space below to take notes) to facilitate these tasks and were given time to review the materials before the afternoon's events. Notes from each working group were collected and synthesized to inform specific actions the Alliance should take when developing their strategic plan.

### Successes, challenges, and lessons learned

Following the retreat, ten participants from varying perspectives and levels of involvement in the development, dissemination, and/or participation in the consensus method were asked to participate in a semi-structured interview. Five were interviewed (six agreed to participate, one withdrew their responses). The interview guide (Supplementary File S2) asked questions about the successes, challenges, and lessons learned from implementing the consensus method.

#### Interview analysis

All interviews were audio-recorded, transcribed, and checked for accuracy by EG. Identifying information was anonymized for each transcript. All transcripts were subjected to a collaborative reflexive thematic analysis (28, 29). EG re-read all transcripts and took detailed notes to familiarize themselves with the data. EG then used the interview transcripts and notes to identify initial codes, which were then constructed into draft themes. EG presented the draft themes to PA, HG, and SS, who in their role as critical friends helped to refine, define, and name each theme (30).

### Consensus method outcomes and perspectives

#### Survey reach

Survey 1 was disseminated to over 2,500 members of the Ontario SCI community, including Alliance members (i.e., researchers, clinicians, policymakers); SCIO staff, volunteers, and membership; and peer activists. Table 1 includes detailed demographics for survey participants. In total, 374 people completed Survey 1 (*Mean Age: 54.8 years, 32% female*); with 78% of respondents *(n = 291)* having SCI lived experience (*24% tetraplegia)* and a response rate of 15%. For Survey 2, 118 people responded (31.6% of Survey 1 respondents) (*Mean Age: 54.7 years, 33% female)*, with 74% of the 118 having SCI lived experience *(24% tetraplegia).* Examples of “other” roles indicated in both surveys included SCI peer mentor, non-profit organization staff or volunteer member, and SCI advocate. Over 70 Survey 2 respondents expressed interest in attending the retreat. For pragmatic reasons (e.g., not all interview attendees who indicated interest in attending came for the event, not all attendees stayed for the entire event), we were unable to collect demographic information from retreat participants. To maintain participant confidentiality, demographic information about retreat participants and interview participants is not presented.

**Table 1:** Demographic characteristics of survey participants.

Characteristic	Survey 1	Survey 2
Number of participants	*n* = 374	*n* = 118
Age	Mean: 54.8 years	Mean: 54.7 years
Gender		
Male	*n* = 255; 68%	*n* = 79; 67%
Female	*n* = 119; 32%	38; 33%
People with SCI	*n* = 276; 74%	87
Paraplegia	*n* = 204; 74%	67; 76%
Tetraplegia	*n* = 71; 26%	20; 24%
Primary role		
Person with SCI	*n* = 187; 50%	*n* = 71; 60%
Family/friend	*n* = 14; 4%	*n* = 3; 3%
Community service Provider	*n* = 26; 7%	*n* = 9; 8%
Hospital clinician	*n* = 11; 3%	*n* = 0; 0%
Community clinician	*n* = 4; 1%	*n* = 2; 2%
SCI researcher	*n* = 7; 2%	*n* = 2; 2%
Funder or policy maker	*n* = 4; 1%	*n* = 0; 0%
Other	*n* = 119; 32%	*n* = 29; 25%

#### Survey & retreat outputs

Using the findings from the survey and retreat, the Ontario SCI Alliance held a series of meetings to further identify community members' strengths in pushing for implementation of the selected priorities. Examples of noticeable changes that were made as a result of the community-based consensus exercise are presented in Table 2 (31–33).

**Table 2:** Examples of implemented changes from the community-based consensus exercise.

Domain	Description of change	Resource/reference
Primary care and community supports	Expansion of primary care for people with SCI, including a reduction in wait times for accessing primary care, and increased accessibility for examination rooms	Centre for Family Medicine Mobility Clinic Team (31)
Pressure injuries	Development of a wound care strategy in partnership with the Ontario government	
Bladder management	Creation and finalization of a campaign for public coverage of intermittent catheters and urinary care supplies	#PeeForFree Campaign (32)
All domains	Implementation of educational toolkits top be used for home and community care	Accessed through the SCIO Cortree Educational Series (33)

#### Successes and challenges of the consensus method

From the reflexive thematic analysis, five core themes were co-constructed to highlight perceived successes and challenges of the consensus method: (1) *Inclusion, Diversity, Equity, and Accessibility (IDEA);* (2) *Partnership;* (3) *Design*, (4) *Transparency and Clarity in Communication; and* (5) *Sustainability*. Supplementary File S3 includes further explanations of each theme supported by interviewee quotes.

#### Inclusion, Diversity, Equity, Accessibility

Deliberate prioritization of making the consensus method more Inclusive, Diverse, Equitable, and Accessible (IDEA) was integral to the perceived success of the method. Intentionally considering *who* was involved in the consensus exercise, and *how* to meaningfully involve people exemplified how the method supported IDEA. For example, placing “decision-makers” (e.g., government policymakers) at each working group table ensured that someone with the power and authority to induce change heard everyone's thoughts. Multiple methods to facilitate participation were also seen as beneficial for promoting inclusive and accessible decision-making practices. For example, individuals unable to attend the retreat could still meaningfully provide input by completing the survey(s). Additionally, providing opportunities to participate through both a survey *and* a retreat, regardless of role or expertise, encouraged the sharing of multiple and diverse perspectives. The Alliance used accessible language in the survey(s), presentation, and reading materials to increase comprehension, added open-ended survey questions to elicit perspectives outside of synthesized evidence, and pre-assigned working group tables to facilitate multidisciplinary interactions. Finally, the importance of including SCI networks and organizations at each stage of the consensus method was mentioned, as networks/organizations can act as a single entity while representing many community members.

A notable challenge interviewees expressed was that the Hart House was not conducive for the retreat. The Hart House had limited wheelchair parking and limited physical space indoors, creating a barrier by preventing more people with SCI lived experience from participating. Overall, interviewees stressed that the unique needs of people with SCI must be known and well-understood when designing an initiative or event that people with SCI are asked to attend. Examples of considerations included: scheduling later start times to accommodate for time needed for self-care and selecting centrally located events for greater public transit options.

#### Partnership

Partnering between academic researchers and a community network was considered critical for designing and delivering the consensus method within a short time frame. Favourable features of the partnership included the evident trust between the academic and community partners and that both partners were always thinking about how decisions would benefit both parties.

Conversely, some interviewees felt the partnership was missing perspectives from industry organizations, which may have limited the retreat's potential. It was mentioned that academics and non-profit organizations may not be as well trained as the for-profit industries in hosting events to share research with diverse audiences. A second challenge interviewees discussed was that timelines and priorities for academia and communities differ and can conflict. Specifically, community organizations' priorities change to reflect the needs of their membership at a rate that may not align with institutional requirements for university-based research projects (e.g., ethics board approvals, time for applications and manuscripts to be reviewed).

#### Design considerations

Incorporating qualitative questions in the survey(s) (i.e., open-ended questions) was praised by interviewees as it allowed respondents to share opinions that did not align with the Likert scale question options. Grounding the development of the survey in Delphi methodology was also praised by interviewees, as it allowed for information to be presented systematically to facilitate simpler decision-making processes. Interviewees highlighted two specific decisions that contributed to the high turnout and level of engagement at the event: location of the event and the use of an external facilitator. The Hart House, located in Toronto, the largest city in Ontario, Canada, acted as a central public transit hub and ensured attendees had several options to get to the event. Interviewees commented that hosting the presentation and panel in a smaller room within the Hart House may have bolstered people with SCI's confidence in asking questions to researchers/clinicians, where a power dynamic is usually present. Finally, hiring an external facilitator to lead the working group tables was highlighted as a strategy to ensure all attendees were comfortable sharing opinions, regardless of their roles or expertise.

Conversely, some interviewees felt that hosting the event at Hart House may have impeded inclusive decision-making processes. Some individuals were unable to attend the retreat due to capacity limits or an inability to get to the event (those from rural/remote communities), meaning these individuals' insights were not heard or included during the working group tables.

#### Transparency and clarity in communication

The need for transparent and clear communication by the partnership to survey and retreat participants was discussed by almost all interviewees as critical for promoting engagement and trust.

Interviewees highlighted how the purpose of the survey(s) and retreat was communicated very transparently to the Alliance members involved in refining the survey, and retreat participants with more active roles (i.e., presenters, panel members, external facilitator, table facilitator(s), note-taker(s)). However, communication about the event's purpose could have been clearer and more transparent to other survey and/or retreat participants. Interviewees also expressed that clearer communication about *who* was involved during each stage of developing the consensus method (e.g., Summit participation, survey development and dissemination, retreat activities) may have promoted more engagement by ensuring people knew whose perspectives informed each stage.

When asked how communication could be improved, interviewees recommended that decision-making could be further simplified if a guiding framework was used to explain to survey and retreat participants the different ways they could be involved in the method (e.g., the Spectrum of P2) (34).

#### Sustainability

The importance of strategizing how to formalize the consensus method was discussed, as formalization would likely allow for the method's use in guiding future decision-making processes for the Alliance. Some interviewees felt the method itself was evidence of shared decision-making at a community level, and that presenting the method and its impact in academic formats (e.g., conference presentations, peer-reviewed journals) would ensure funding organizations viewed the method as rigorous and evidence-based. Involving a graduate student trainee was also viewed as critical in ensuring the sustainable use of the consensus method. Involving trainees as co-leads in the partnership ensured that the values of partnering and meaningful engagement are instilled early in one's career, and maintained as trainees transition into independent researchers.

An inability to maintain the same level of communication with participants *after* the retreat, as was done *during* the surveys, was viewed by interviewees as a challenge for sustaining the engagement and impact of the method. An effort that could have been undertaken to promote sustainability included updating the membership on how the selected strategies are being implemented. However, interviewees also indicated that ensuring receipt and understanding of these updates by participants would be difficult to monitor and address.

## Discussion

Our academic-community partnership co-designed a community-based, consensus method to harness the opinions and perspectives of the Ontario SCI community to inform research and policy-based decisions. The method consisted of two modified Delphi surveys and one in-person retreat; all of which were well attended by members of the Ontario SCI community, in particular people with SCI lived experience. Following the retreat, the Ontario SCI Alliance synthesized the survey and retreat materials to inform their strategic plan and deliver relevant policy changes for their membership. Five themes around successes, challenges, and lessons learned from the method were co-constructed from our collaborative reflexive thematic analysis: (1) *Inclusion, Diversity, Equity and Accessibility* (*IDEA*); (2) *Partnership*; (3) *Design Considerations;* (4) *Transparency and Clarity in Communication*; and (5) *Sustainability*. These themes highlight key factors to consider when co-designing and implementing a community-based consensus method.

### Survey development and dissemination

Modifying our approach rather than adhering to the prescriptive criteria of traditional Delphi exercises aligns with previous efforts of research users with differing needs and priorities to arrive at consensus (35, 36). Including a retreat was similar to previous modifications (e.g., online discussion, workshops, focus groups). However, modifying the survey questions and incorporating qualitative, open-ended questions is less common practice. This intentional decision helped facilitate inclusion and break down knowledge hierarchies by allowing for anecdotal, lived experience to be considered with the same weight as evidence-informed strategies during decision-making. Considering that attention to inclusion has rarely been noted in the consensus literature (10), this reproducible strategy can support researchers and communities to promote inclusion during consensus exercises.

Preparing and conducting consensus methods in partnership is not novel, but partnership guidance for groups to refer to remains limited (10). Delphi guidance primarily targets traditional methods, and internal modifications to consensus methods are rarely reported with transparency or detail, making reproducibility and an understanding of participants' roles in a Delphi difficult. To address this qualm, we transparently report on our method's preparation, conduct, and analysis through our diverse partners' perspectives (Supplementary File S1) and highlight challenges that arose (e.g., tight timelines for funding applications). We hope providing highly detailed and transparent reporting may help normalize the reporting process, make modified Delphi exercises more reproducible, and provide valuable information that can be used to develop and evaluate acceptable criteria for modified Delphi exercises.

Previous guidance recommends six “expert” participants as the requirement for a Delphi to be a reliable consensus method (37). While 374 participants exceeded this recommendation, our overall response rate was only 15% as the survey was disseminated to over 2,500 people. Seventy percent is suggested as a desirable rate for maintaining rigour in a Delphi exercise, though this guidance is specific to a Delphi with 6–30 experts (38, 39). Previously established Delphi recommendations may not be appropriate for community-based Delphi methods, given the distinct differences in the number of participants. Future research should focus on expanding and evaluating Delphi criteria to accommodate and engage more participants.

Booking the retreat at a small venue facilitated more intimate conversations. However, this decision ran the risk of tokenizing the people with SCI in attendance, particularly if efforts were not undertaken to mitigate power dynamics during decision-making conversations (40). Since our retreat, the COVID-19 pandemic has normalized virtual/hybrid engagement efforts, which would likely simplify implementing virtual engagement supports to accommodate for travel restrictions and varying levels of comfort with in-person interactions. Future studies should explore if and how virtual engagement changes any of the method's outcomes or impacts.

### Successes, challenges, and lessons learned

Prioritizing IDEA throughout the stages of the consensus method fostered meaningful participation from diverse perspectives of the Ontario SCI community. Certain strategies adopted by our team have previously been used to mitigate power dynamics and maximize participation throughout the consensus process, including external facilitators (41, 42) and scheduling the retreat alongside an annual meeting (43). Our findings add to the literature by suggesting specific strategies for promoting meaningful engagement with people living with SCI (e.g., using surveys to prevent inflexible participation times; later start times to accommodate for self-care tasks) that can guide researchers and policymakers to make their processes more inclusive and accessible for people with SCI specifically. However, we caution that adopting these strategies does not automatically translate into *full* inclusion, diversity, equity, and accessibility for people with SCI. Rather, the strategies can be undertaken to adopt *more inclusive, accessible, equitable and diverse* practices. Full “IDEA” cannot be achieved, as understandings of IDEA are different and even conflict with one another, based on one's unique intersectional identity and environment (44, 45).

Attribution of the method's success to a strong academic-community partnership is not unexpected, as the science of research partnerships has advanced since our work in 2018. Published in 2021, a multidisciplinary panel of SCI researchers, research users, and funders rigorously co-developed the Integrated Knowledge Translation Guiding Principles for Conducting and Disseminating Research in Partnership (4), which outline eight values for all research partners to follow early and throughout the research process. Our findings can further advance the science and use of these principles by proposing observable, actionable strategies that can support partnerships to follow specific principles (Table 3). Additionally, our findings demonstrate how SCI research partnerships can integrate policy-making with research. Future research efforts should focus on how principles and strategies for policy-focused partnerships may differ from the current research partnership literature.

**Table 3:** IKT guiding principles with example strategies adopted by our partnership.

Principle	Example of strategy
1. Partners develop and maintain relationships based on trust, respect and dignity	PA and HG have a previously developed partnership that began in 2013. Through transparent communication and regular check-ins with on another, trust was developed between them, allowing them to engage in a second research project within their partnership.
2. Partners share in decision-making	Decisions at each stage of the project were made collectively by all partners, and the project did not move forward unless all partners had a meaningful say in the final decision (see Supplementary File S1).
3. Partners foster open, honest, and responsive communication	All partners were aware of each other's preferred communication methods and engaged in all project communication using these methods to ensure responsiveness.
4. Partners recognize, value, and share their diverse expertise and knowledge	PA's unique and extensive knowledge of SCI through his lived experience and time working with SCIO and the Alliance was shared with all partners, and incorporated throughout the methods (e.g., which domains to include in the survey, who to disseminate the survey to).
5. Partners are flexible and receptive in tailoring the research approach to match the aims and context of the project	Changes were made to the Delphi method protocol to match the aim and context of the project Aim: To ensure that this was a “community-based” Delphi, the number of “experts” was not limited in terms of the number of participants, or their “knowledge/expertise” in the subject. Context: Traditional timelines for the Delphi were modified to accommodate for the Alliance's deadline in creating their strategic plan.
6. Partners can meaningfully benefit by participating in the partnership	Academic partners: outputs of this project have aligned with indicators for academic merit (a successful grant application, 2 poster presentations at academic conferences, 1 peer-reviewed manuscript, 1 national trainee award). Community partners: outputs of this project resulted in an evidence base that could be used to inform the Alliance's upcoming decisions for research implementation over a 3 year period.
7. Partners can address ethical considerations	Given the unique roles within the Ontario SCI community, it can be easy to identify participants’ throughout the Delphi. Throughout surveys and interviewing, all partners strategized how we can maintain participant confidentiality while meaningfully disseminating results.
8. Partners respect the practical considerations and financial constraints of all partners	The MITACS funding opportunity was selected to fund this project as it supported the Alliance (a non-profit entity) to hire a trainee to complete work without impacting the organization's operations, while ensuring EG was compensated appropriately for her time and efforts.

Our third theme, Transparency and Clarity in Communication, aligns with Principle 3 of the IKT Guiding Principles: *Partners foster open, honest, and responsive communication.* Considering only challenges were discussed within this theme, it is likely that our interview participants greatly value strong communication within and beyond the partnership, and wanted to ensure that efforts to improve communication were vocalized. Potential reasons that communication to participants may not have been as meaningful include the tight timeline for designing and conducting the method in order to accommodate the Alliance's needs. Previous scholars have emphasized the importance of recognizing the time and effort needed to execute a consensus method in a meaningful and engaging way (43). In thinking about meaningful communication with people with SCI, previous research has examined preferred communication methods for other topics, such as physical activity messaging (46) and peer mentorship (47). Similar efforts to understand preferred communication methods and timelines are likely needed to improve policy-based decision-making with people with SCI.

A critical future direction from our work is involving trainees as co-leads in research partnerships to normalize and motivate others to partner meaningfully with equity-deserving groups. We provide a detailed account of a trainee's capacity to hold a leadership role in a research partnership that also explains strategies for addressing issues that arose. Our work adds to previous efforts by Nguyen and colleagues regarding trainee involvement in partnerships (e.g., it is okay to not know what a partnership looks like; there is no single recipe for how to partner; take time to invest in partnerships; provide ongoing opportunities to reflect; consider balancing power dynamics and incorporating diversity) (48). EG was also awarded the national Mitacs Award for Outstanding Innovation as a Master's student for this project, suggesting that funding bodies are prioritizing partnerships with equity-deserving communities. Our work can help to inform efforts to advance the capability of trainees to partner with equity-deserving groups.

### Strengths and limitations

A notable strength is the absolute number of individuals who participated in the surveys and retreat. To our knowledge, this is the highest number of individuals with SCI to meaningfully participate in a Delphi for making policy-based decision-making. Second, conducting the consensus method in partnership allowed for research, clinical, policy, and SCI lived experience perspectives to be meaningfully incorporated while designing the survey(s) and retreat, which has helped to increase the relevance of the strategies included in the surveys, and strategies discussed during the retreat.

While our partnership did follow other evidence-based strategies to improve response rates, such as sending reminder e-mails, additional efforts could have been undertaken to improve response rates, such as clearer explanations of the study process and the importance of commitment throughout the surveys and providing incentives for completing surveys (38, 39). Second, as we were unable to collect demographic data on retreat participants, we cannot make any inferences regarding how attendees' demographics may or may not have impacted their retreat experiences. Third, e.g., delivered the presentation at the retreat. Given their role as a trainee, EG may have been uncomfortable probing further into negative comments about the consensus method, or interviewees may not have wanted to share negative thoughts with EG Fourth, our conceptualizations of IDEA were framed by individuals and organizations working to combat ableist societal views. As such, our proposed strategies for promoting IDEA cannot be assumed to address *all* systems of inequity (e.g., sexism, racism, etc.). Future efforts to implement and evaluate consensus-based methods should adopt an intersectional lens to ensure that multiple inequities are considered when developing strategies to promote IDEA. While not a methodological limitation, the retreat occurred before the COVID-19 pandemic, and any claims from our findings that signify the importance of using in-person methods were not made with knowledge of the pandemic.

## Conclusion

Our academic-community partnership co-developed a community-based consensus method that meaningfully engaged a large SCI community in determining research and policy decisions. While there are still challenges to address, our detailed account of the development, dissemination, and execution of the method can support other organizations/networks that represent equity-deserving groups to meaningfully engage those with lived experience in their decision-making processes.

## Data Availability

Raw data will be made available upon reasonable request.
