# Supplementary material for: A case study of using community-based consensus methods to facilitate shared decision-making among a spinal cord injury network
Source: Front Rehabil Sci. 2024 Feb 16;5:1335467. doi: 10.3389/fresc.2024.1335467 (PMC10904660; doi:10.3389/fresc.2024.1335467)
Supplement: Supplementary file 1 [file Table1.docx]

**Supplementary File 1: Detailed Log of Partnership Activities (Survey and Retreat)**

| **Research Phase** | **Date** | **Strategy/Activity** |
| --- | --- | --- |
| Planning | Sep 2017 | PA approached HG in an initial discussion about an opportunity that the Ontario SCI Alliance wanted to undertake to ensure their upcoming strategic plan was developed in partnership with the broader SCI community.  Given EG’s connection to the Ontario SCI community, HG (EG’s supervisor) proposed that EG be the lead trainee for this opportunity. |
| Planning | **Early Oct 2017** | Based on the needs of the project, HG recruited SS to join the partnership, given their expertise in research methodologies and working with communities. |
| Planning | **Writing:**  Oct – Nov 2017  **Funding Approved:** Dec 2017 | Initial conversations were had between HG, PA, SS, and EG, where PA shared what the Ontario SCI Alliance’s goal was (i.e., to ensure that strategies for inclusion in their strategic plan were informed by their membership). Using their knowledge and expertise in research methodologies, HG and SS proposed that a consensus method be the best approach to address the Alliance’s aim. EG prepared for their involvement by reading and reviewing consensus literature (SCI and non-SCI specific).  HG and PA co-wrote an application for funding to MITACS Canada, which was reviewed by SS and EG prior to submission. |
| Survey 1 Development | **Initial Draft Started:** October 18, 2017  **Initial Draft Completed:** November 9^th^, 2017 | PA shared an initial document with the partnership outlining strategies that the Ontario SCI Alliance was interested in implementing.  EG was then tasked to create an initial draft of a Delphi survey that was sent back to PA, SS, and HG for review until the partnership was confident in the draft. Examples of changes implemented to the survey:   - PA recommended the use of open-ended questions to ensure that people’s ideas were not limited to the strategies previously developed from the Summit meetings. - HG and SS advised on the use of an 11-point Likert Scale for the Delphi surveys, and randomizing the order for which strategies were presented in. - Following SCIO’s guidance, all strategies were re-worded to align with a Canadian Grade 8 reading level (~Age 12-13) to accommodate for all levels of education for participants. |
| Survey 1 Development | Nov 13^th^, 2017 | PA shared the initial working draft version of the survey with the Summit Leads for each included domain (one clinician per domain). All domains were approved by the Summit Leads, with minor feedback regarding formatting provided. |
| Survey 1 Development | Nov 30^th^, 2017 | Meeting between EG, PA and funders of the Ontario SCI Alliance (i.e., staff from SCI Ontario, Ontario Neurotrauma Foundation, Rick Hansen Institute*) to present the initial version of the survey.  Examples of additional roles held by funders included people with lived experience, researchers, clinicians, and SCI community-based organization executives.  Proposed changes were sent back to EG to implement to the initial working draft version of the survey. All changes were mostly minor, related to formatting and wording. |
| Survey 1 Development | Dec 1^st^, 2017 | EG shared the survey with 4 members of the Ontario SCI community to pilot for understandability and accessibility. |
| Survey 1 Development | Dec 4^th^, 2017 | The updated version of the survey was shared with HG, PA, and SS for additional feedback. All changes were related to formatting, wording, and ensuring the context was appropriately addressing the Ontario SCI community’s needs.  Once remaining changes were implemented, survey was finalized and ready to be launched. |
| Survey 1 Dissemination | Dec 7^th^, 2017 | Survey was internally launched via e-mail to SCIO Staff. |
| Survey 1 Dissemination | Dec 11^th^, 2017 | Survey was externally launched via e-mail and social media to the Ontario SCI Alliance Membership. |
| Survey 1 Dissemination | December 2017 – January 2018 | Reminder e-mails were sent two weeks, one week and one day prior to the survey closing.  All templates for reminder e-mails were drafted by EG, and reviewed by HG, SS, and PA for additional feedback. |
| Survey 2 Development | January 9^th^, 2018 | To accommodate for short timeframe for analyzing Survey 1 and creating Survey 2, EG began drafting the descriptions and definitions for Survey 2 to align with Delphi methodology for review by PA, SS, and HG. No changes were made to descriptions/definitions of the strategies/domains, as these descriptions were pre-approved by Summit Leads, Alliance Funders, and Ontario SCI community members during initial piloting.  Changes proposed included:   - Including an option for raters to indicate their top priority (in the case that all strategies receive a high rating) to help complement the scores. All partners agreed to this suggestion. |
| Survey 1 Dissemination | January 19^th^, 2018  February 2^nd^, 2018 | Initial Survey 1 Closing Date: January 19^th^, 2018  Revised Survey 1 Closing Date: February 2^nd^, 2018   - A decision was made and agreed upon by the partnership to close the survey at a later date, given the initial survey was sent over the holiday season. This would allow for more opportunities to respond for those who were not actively checking e-mails or social media over the December holiday season. |
| Survey 1 Analysis | Feb 2^nd^ – Feb 14^th^ | Under the supervision of HG and SS, EG began analyzing the data from Survey 1. While analyzing, EG would check-in regularly with PA (~2x per week) to update him on the analysis, and check to see if there were any additional ways that the data should be analyzed to ensure relevance for the Alliance. |
| Survey 2 Dissemination | February 14^th^, 2018 | Link to complete Survey 2 was sent over to Survey 1 participants who consented for further participation. |
| Survey 2 Dissemination | March 7^th^, 2018 | Survey 2 Closing Date |
| Survey 2 Analysis | March 7^th^ – 29^th^, 2018 | Under the supervision of HG and SS, EG began analyzing the data from Survey 2. While analyzing, EG would check-in regularly with PA (~2x per week) to update him on the analysis, and check to see if there were any additional ways that the data should be analyzed to ensure relevance for the Alliance.  During this time, PA was continuing to connect with the Alliance’s funders to keep them updated on how data analysis was going, and relayed all changes. |
| Retreat Design | March 27^th^ , 2018 | PA introduced an external facilitator to the partnership, and shared his first draft of the facilitation design of the event for review by HG, EG, SS, and PA. |
| Retreat Design | March 29^th^, 2018 | EG connected with PA’s policy staff to begin creating communication materials for the upcoming Ontario SCI Alliance Retreat, which were inclusive of the survey results. |
| Retreat Design | April 4^th^, 2018 | External facilitator shared final copy of facilitation plan (with incorporated feedback) with PA, who further shared with HG, SS, and EG for opportunities to provide final feedback. |
| Retreat Design | April 12^th^, 2018 | EG, PA, SS, and HG put together the communication materials required for the Retreat into binders (e.g., agenda, summary of survey findings, worksheets for facilitated discussion). |
| Retreat Conduct | April 13^th^, 2018 | Ontario SCI Alliance Retreat |

*Note: Rick Hansen Institute is now referred to as the Praxis Spinal Cord Institute*
