# Supplementary material for: A case study of using community-based consensus methods to facilitate shared decision-making among a spinal cord injury network
Source: Front Rehabil Sci. 2024 Feb 16;5:1335467. doi: 10.3389/fresc.2024.1335467 (PMC10904660; doi:10.3389/fresc.2024.1335467)
Supplement: Supplementary file 2 [file Table2.docx]

**Supplementary File 2. Interview Guide**

**Part 1: Introduction**

Thank you so much for your interest in participating in this study which aims to evaluate the two surveys sent out earlier this year by the Ontario SCI Alliance., as well as the Ontario SCI Alliance Retreat.

Your participation in this study is completely voluntary and you are free to not answer any questions if you are uncomfortable doing so. You can let me know if you would like to withdraw at any point. Additionally, you can e-mail Dr. Gainforth to withdraw after completion of the interview and have your data removed from the study.

**Part 2: Consent & Participant ID Code**

Please confirm that you consent to participating in this study, knowing that you can withdraw at any time with no consequence to you.

Please confirm that you consent to having this conversation recorded.

To create your unique participant ID code, please answer the following three questions. This code will protect your confidentiality when linking responses to demographic data:

- What is your mother’s maiden name?
- What are the two digits of your date of birth?
- What are the final two digits of your telephone number?

**Part 3: Interview Questions**

The questions that will be asked throughout this interview will be particular to the two priority-setting surveys that were distributed by the Ontario SCI Alliance, as well as the Retreat which took place on April 13^th^, 2018.

| **Background Questions** | Please describe your role as a participant of this initiative (I.e., for the surveys and/or Retreat), or the Ontario SCI Alliance overall.  Why were you interested in being part of the survey and Retreat? |
| --- | --- |
| **Experience Questions** | What was your overall experience throughout this initiative?   - ***Probe:*** Specific to the Alliance survey(s)? - ***Probe:*** Specific to the Retreat?   Did this experience demonstrate equal participation between people with spinal cord injuries, service providers, policy makers, and researchers? Why or why not? |
| **Success Questions** | What do you think were the overall successes of this Alliance initiative?   - ***Probe:*** Specific to the survey(s)? - ***Probe:*** Specific to the Delphi method? - ***Probe:*** Specific to the Alliance Retreat? - ***Probe:*** Was the morning presentation helpful in decision-making for the afternoon? |
| **Challenge Questions** | What do you think were the overall challenges of this Alliance initiative?   - ***Probe:*** Specific to the survey(s)? - ***Probe:*** Specific to the use of a Delphi method? - ***Probe:*** Specific to the Retreat? |
| **Recommendation Questions** | Do you have any recommendations to make if the Alliance were to undertake a similar initiative in the future?   - ***Probe:*** Specific to the survey(s)? - ***Probe:*** Specific to the Retreat? |
| **Concluding Questions** | Is there anything else you would like me to know that may have been missed in any of the questions above? |
