# Supplementary material for: A case study of using community-based consensus methods to facilitate shared decision-making among a spinal cord injury network
Source: Front Rehabil Sci. 2024 Feb 16;5:1335467. doi: 10.3389/fresc.2024.1335467 (PMC10904660; doi:10.3389/fresc.2024.1335467)
Supplement: Supplementary file 3 [file Table3.docx]

**Supplementary File 3.** Exemplary Quotes for Co-Constructed Themes

| Name of Theme | Code | Interview Excerpt |
| --- | --- | --- |
| IDEA | Intentionally considering who was involved & how to meaningfully involve them | “And I think it provided equal footing for everybody for the discussion and debate. I thought that, as we moved into the latter part of the day and the sort of breakout sessions that were developed, I thought there was a good balance of individuals. The table assignments created a really interesting discussion while having different stakeholders at different tables coming from a different background. I thought that was a very useful tool that you guys utilized.” |
|  | Encouraging multiple perspectives through survey and retreat participation (having multiple methods for participation) | “I'm very happy that we accumulated multiple perspectives of different stakeholder groups from a quantitative manner and a qualitative manner, and I've said earlier that I enjoyed the qualitative responses more than the quantitative responses because they were very real, they were very personal. It showed struggle and it showed vulnerability, it showed what the expectation is from the Alliance and the partnering organizations and what does success look like from an individual point of view and I feel that we at times move forward with the best information we have and exclude the emotion associated with the urgency of making change and I think we capture the urgency and the expectation very well.” |
|  | Information needs to be accessible to everyone | “So I thought the presentations were excellent in terms of bringing people up to speed. So if there were people that were not part of everything. By the end of the presentations. There was clarity around what would have been achieved, what to move forward, So I thought we sort of, kind of the survey that sort of what people had talk about, was brought to people so that everyone have a common understanding by the time we went into the workshop.” |
| IDEA | Hart House | “So some choices that we've made compromise some accessibility in terms it was harder for people with disabilities to get around in the rooms and we also took a very historical approach in hosting an event in a very historic place where change happened throughout Canada right we chose the heart house in the debates room where are you united are our first Nations built the first treaties of Canada before Confederation and purposely chose that theme to show the growth of where we've come and where we want to go and how us as a community have to maintain the moral fabric of our country.” |
|  | Considering unique needs | “I think the major challenge is that you know we put such large expectations on our SCI community. You know we recognize that our community has full-time jobs, full lives, children, their own pressures, their own personal challenges and things that they are focused on and then we continuously try to seek their opinion on a variety of different manners and you know as much as we like to get the largest voices and the larger sample sizes of how we wanted to gather information.”  “And in many ways, if you didn't have partners that were invested on both sides, it wouldn't have happened. So, you needed an organization that was invested and believed in the process. You needed a motivated, enthusiastic student that was willing to do it. And you could have ended up with a student that wasn't like that. And without that, it would have been probably next to impossible. And then, you needed a research team that was willing to work on tight timelines and prioritize this project and the timelines of this project.” |
| Partnership | Trust | “I was interested because I have a longstanding relationship with Spinal Cord Injury Ontario and the alliance, and I've been working with them for a number of years. So as a result, I was invested in the success of the organization. And at the same time, it also aligned with my values and goals in the sense that I'm really interested in ensuring that research is used in practice and ensuring that, when we make decisions about implementation, people's voices are heard and that there's nothing about people with disabilities without them. And so, as a result, these methods help to actually do that.” |
|  | Reciprocal Benefits | “Well I integrated [the exercise into] an ongoing system, so in fact if we are going to do something, how does that fit into the broader piece, so we’re only one component [that] allows that to sort of move forward that actually makes my job a lot easier and it also sets a pattern so that if we are to come up with a result out of some intervention, of where’s it [going next] so it doesn't just sit on the shelf and then again, you know publication is an important piece [of dissemination], but it’s not the only piece. So, people have to do that but you know we shouldn’t concentrate on just that, so how would we bring that into the force if we were looking for that.”  “I'd like to believe that the students that have been involved in this project are actually getting something of this project and are using it towards their careers. And if we can create that environment. It just creates a whole whack of successes everywhere.” |
| Partnership | Industry Perspective was missing | “That's why you'll see some of the private sector colleagues […] they actually have warehouses where they actually do [these types of exercises that are more novel to researchers]. The whole thing is walls. There's nothing but walls that auto-record. They have entire data processing in the back end. There's stuff coming back in the group in their format. "What about that thing you wrote on the corner? Oh, there it is." |
|  | Academic and Community timelines do not align | “And in many ways, if you didn't have partners that were invested on both sides, it wouldn't have happened. So, you needed an organization that was invested and believed in the process. You needed a motivated, enthusiastic student that was willing to do it. And you could have ended up with a student that wasn't like that. And without that, it would have been probably next to impossible. And then, you needed a research team that was willing to work on tight timelines and prioritize this project and the timelines of this project.” |
| Design | Open-ended questions (qualitative questions) | “Instead of presenting options to them about, "Would you rather have this treatment or this therapy?" to talk about what challenges or what obstacles they're facing in accessing treatments or therapies, and that would have, perhaps, brought out answers along, "I have difficulty accessing the wound care team," or, "I had a wound, and they utilized this treatment, and it was not effective for me. I found out another person had an opportunity to use this other treatment which was more effective. I wish I would have had access to that treatment," and in that way, you could start to narrow down where the barriers or issues were, that it was a question of access, or it was a question of treatment options, or it was a question of knowledge of the practitioner, as opposed to identifying what the potential problems are and saying, "Would you rather this or rather that?" |
| Design | Systematic Delphi |  |
|  | Location (Hart House) |  |
|  | External Facilitator | “I do, I think people were not shy about sharing their opinions, I think there was an equal playing field, there was not any one particular group that sort of dominated or lead or sort of got very clinical so people couldn't understand the vocabulary cause people talk in code or got overly policy oriented, so I thought and it was very much pragmatic, so my sense that sort of in the groups I sat in, I don’t think anyone felt they were not, and the facilitator did a great job making sure everyone’s comments and voices were heard.” |
|  | Location (Hart House) | “Yes. I think one of the biggest challenges when you're doing these types of events when you're working with a venue, the venue has to be involved a lot sooner than most people think. People usually book a venue, and then when they get close to the venue they start talking to the venue. Usually, you have to talk to the venue. You have to involve the venue in the plan so they get a sense of the flow. One of the things that a lot of people now do is they do a 24-hour prior preview. They walk through every step of the event 24 hours in advance with all the people involved. That's the best practice they can do. It's called a dress rehearsal.” |
| Communication | Clearer to those involved, less clear to others | “Exactly, and where they were seeking input and influence. I think a few of the attendees seemed to think that there was more of a “blue sky” exercise going on, where you would have opportunity to direct the activities a little bit more broadly as opposed to within the narrow context of the priorities that are even pre-identified before the meeting.” |
| Communication | Who was involved needs to be more transparent | “So, we build a profile when we say community, who that community is that was actually involved in that external interviewing process. So, the people inside of the room get a mental picture of who are these other larger groups that we're talking about. When the survey was done, did the overview in the morning. I'm just saying, sometimes it helps to get right down into who is the larger group participating.”  “I think perhaps, as I said earlier, the scope of influence was what became clearer as the day went on, and I think that could have been made more clear earlier in the day.” |
| Communication | IAP2 & frameworks | “Well, I would recommend googling [the] IAP2 Spectrum of Engagement. It's a fantastic little tool that can be utilized when you're engaging the public for any type of interaction, really. It lets you know-- it helps you to define what your objectives are, and you can make your participants aware of where their scope of influence truly lies, and it creates a really transparent environment where you can really-- it allows people to know where they stand. There's inform, there's consult, there's empower, and none of those are wrong. So, you can utilize those tools in different ways. So, like I was saying about the final report, right, so that would be inform on that spectrum. You're not seeking any feedback whatsoever. You're just making people aware of what you're doing behind the scenes, and then, when you send them that final report, you're probably not looking for any feedback on that report. You just want to send that out to inform people that they're there, right? For the actual meeting on the day, I think a lot of people felt that this was a consultation as consults fits in this, but really, I think it was more of an involvement, where people were asked their opinions, but there's really no promise from the Alliance that they would be beholden to the things on the day, whereas we focused more on the consultation side, where power thoughts would have a little impact on the decision-making process.” |
| Sustainability | Publishing the method | “We’re evaluating what we’re doing so we can get better at it and that's part of the process. We’re then publishing what we’re doing so we can share the way we do business with the rest of the world because we strongly believe we have strong methodologies of success, and we want to be able to spread those successes across other domains.” |
|  | Sustainability means Involving younger researchers | “And then, invest in enthusiastic students because, when you have students that are keen and eager, they get an amazing experience from coming to work within [SCI Ontario and the Ontario SCI Alliance]. And at the same time, the organization gets somebody that is trained by them and with them. And so, when that student graduates, they get a better resource moving forward in addition to their existing partnerships. So, what you're doing is you're building the partnership pool when you invest in students and younger trainees.” |
|  | Post-event engagement is just as important as pre-event engagement | “Yeah. About the engagement process, right, [the partnership] did a great job of early engagements and making sure people were involved from the get-go of the multiple stakeholder groups. I think one of the problems a lot of groups face is the post-engagement and keeping people aware of your activities and letting them know what the value of their contribution was. So, yeah, but that's one thing I don't-again, you might be preparing to send out an email tomorrow. I don't know. But some post-event follow-up with a clearly defined timeline around what the next steps are is always useful.” |
